# Metabolic effects and pharmacokinetics of oral cannabidiol (CBD) in Connemara ponies following 21 days of treatment

**DOI:** 10.3389/fvets.2026.1813917

**Published:** 2026-05-14

**Authors:** Kata Wermer, Róbert Berkecz, István Ilisz, Dezső Csupor, Nóra Ágh, Anita Sztojkov-Ivanov, Dániel Cserhalmi, Ákos Jerzsele, Orsolya Korbacska-Kutasi

**Affiliations:** 1 Department of Botany, University of Veterinary Medicine Budapest, Budapest, Hungary; 2 Institute of Pharmaceutical Analysis, Faculty of Pharmacy, University of Szeged, Szeged, Hungary; 3 Department of Forensic Medicine, Albert Szent-Györgyi Health Centre, Szeged, Hungary; 4 Faculty of Pharmacy, Institute of Clinical Pharmacy, University of Szeged, Szeged, Hungary; 5 Institute for Translational Medicine, Medical School, University of Pécs, Pécs, Hungary; 6 HUN-REN–PE Evolutionary Ecology Research Group, University of Pannonia, Veszprém, Hungary; 7 Behavioural Ecology Research Group, Center for Natural Sciences, University of Pannonia, Veszprém, Hungary; 8 Institute of Pharmacodynamics and Biopharmacy, Faculty of Pharmacy, University of Szeged, Szeged, Hungary; 9 Budapest University of Economics and Business, Budapest, Hungary; 10 Department of Pharmacology and Toxicology, University of Veterinary Medicine Budapest, Budapest, Hungary; 11 Department of Animal Nutrition and Clinical Dietetics, University of Veterinary Medicine Budapest, Budapest, Hungary

**Keywords:** cannabidiol, cannabinoid, equine endocrinology, equine metabolic syndrome, horse, insulin dysregulation, metabolism, oral sugar test

## Abstract

**Introduction:**

Cannabidiol (CBD) has gained interest in equine medicine due to its potential therapeutic effects. Preclinical studies suggest that CBD may influence metabolic pathways relevant to metabolic syndrome, which present in both human and equine populations. The aim of this study was to evaluate the effects of oral CBD on metabolic parameters in Connemara ponies and to characterize its pharmacokinetics.

**Methods:**

A total of 13 Connemara ponies [seven with equine metabolic syndrome (EMS)] were stratified by EMS status and age and randomly assigned to a CBD-treated group (2 mg/kg orally, twice daily for 21 days; *n* = 7) or a control group receiving flaxseed oil (*n* = 6). Body weight, body condition score (BCS), and cresty neck score (CNS) were recorded pre and post-treatment. Oral sugar tests were performed to assess insulin and glucose responses. Blood samples were analyzed for glucose, insulin, triglycerides, and CBD pharmacokinetics. Safety and clinical parameters were monitored throughout.

**Results:**

CBD did not significantly affect body weight, BCS, CNS, blood glucose, or triglycerides. Insulin concentrations during the oral sugar test increased significantly in the CBD group (mean change: 10.74 ± 5.19 μIU/mL; *p* = 0.046), whereas no significant change was observed in control (−6.21 ± 6.14 μIU/mL; *p* = 0.319). Plasma CBD and metabolite concentrations were measurable, with C_max_ increasing from 22.79 ± 13.24 ng/mL to 39.93 ± 14.45 ng/mL after the, t_max_ ranging from 3.14 ± 1.07 to 2.29 ± 0.76 h, and an accumulation ratio of 2.75 ± 1.51, indicating moderate accumulation. Steady-state was reached within 4–5 days, and treatment was well tolerated.

**Conclusion:**

CBD did not improve metabolic parameters but was associated with an increased insulin response during the OST, suggesting modulation of insulin dynamics of unclear clinical relevance. This finding may raise concerns regarding CBD use in EMS-prone horses. CBD showed predictable pharmacokinetics, with slow elimination and potential accumulation of CBD and 7-COOH-CBD. These findings highlight the need for further studies to clarify dose-dependent metabolic effects and to establish safe and effective dosing strategies, particularly in EMS-prone horses.

## Introduction

Cannabidiol (CBD) is a non-intoxicating cannabinoid derived from *Cannabis sativa* L. with central nervous system activity, lacking the intoxicating and euphoric effects associated with delta-9-tetrahydrocannabinol (THC) (1). The major metabolites of CBD are 7-hydroxy-CBD (7-OH-CBD) and 7-carboxy-CBD (7-COOH-CBD). Although the precise mechanisms of action are not yet fully understood, accumulating evidence suggests that its effects are mediated through multiple components of the endocannabinoid system (ECS), a key regulator of physiological homeostasis (2). Originally defined by cannabinoid receptors CB1 and CB2, endogenous ligands, and metabolic enzymes (3), the ECS is now recognized as a broader signaling network (“endocannabinoidome”) that includes additional receptor families such as peroxisome proliferator-activated receptors (PPARs), serotonin (5-HT), opioid, G protein–coupled receptors (GPRs), and transient receptor potential (TRPs) channels—as well as endocannabinoid-like molecules (4–7). In the liver, cannabinoids undergo CYP450-catalyzed hydroxylation or oxidation, followed by glucuronidation mediated by UDP-glucuronosyltransferase (UGT) enzymes (3).

CBD has been reported to exhibit a range of pharmacological effects, including analgesic, antidiabetic, antipsychotic, antidepressant, anxiolytic, anticonvulsant, antioxidant, and anti-inflammatory activities (4–6). In equine medicine, CBD is most commonly marketed for managing pain associated with osteoarthritis and laminitis, reducing stress, and addressing stereotypic behaviours (7–11). More broadly, CBD and other hemp-derived compounds have been increasingly investigated in veterinary species for their potential to influence physiological and behavioural processes, including pain modulation, inflammation, and stress responses (12). Similarly, plant-derived bioactive compounds, including hemp-based constituents, have been suggested to modulate physiological and metabolic processes in equids beyond their pharmacokinetic profiles (13). Despite this commercial interest, rigorous scientific studies evaluating its effectiveness are largely absent, and the existing evidence is limited or anecdotal at best (14).

In modern veterinary medicine, Equine Metabolic Syndrome (EMS) has emerged as a major clinical concern and a frequent cause of the painful, often life-threatening condition laminitis. EMS is not a distinct disease but a constellation of risk factors that predispose horses to hyperinsulinemia-associated laminitis (HAL) (15). It’s defining and most consistent feature is insulin dysregulation, characterized by disturbances in insulin, glucose, and lipid homeostasis, including basal hyperinsulinemia, exaggerated or prolonged insulin responses to carbohydrate challenges, impaired glucose tolerance, and insulin resistance. Hypertriglyceridemia may develop secondary to insulin resistance. EMS is commonly, though not invariably, associated with obesity, manifested as generalized or regional adiposity and resistance to weight loss. Laminitis is the primary clinical consequence of EMS, although affected horses may also be predisposed to other metabolic complications, including hyperglycemia and hypertriglyceridemia in critical care settings (16). Ponies are more prone to develop EMS than standardbreds (14).

EMS was termed to reflect its similarity to human metabolic syndrome (MetS), a cluster of risk factors linked to coronary heart disease and type 2 diabetes (14). Emerging evidence indicates that CBD may affect several biological processes associated with key components of MetS, including obesity, insulin resistance, dyslipidemia, and non-alcoholic fatty liver disease (NAFLD) (17). Given the parallels between MetS and EMS, including insulin resistance, obesity, and dyslipidemia, it is biologically plausible that CBD could influence metabolic pathways relevant to EMS. However, despite these similarities, important species-specific differences in metabolic regulation, pharmacodynamics, and disease manifestation must be considered when extrapolating findings from human studies to equids. Although preclinical and some clinical studies suggest that CBD may improve glucose regulation, lipid profiles, blood pressure, and liver health in humans, evidence remains limited, and no data currently exist for horses, underscoring the need for further research to establish its efficacy and safety (17). Importantly, EMS and its associated conditions, such as laminitis, have significant welfare implications, and while adjunctive therapeutic approaches may be explored, long-term dietary management and controlled exercise remain the cornerstone of effective disease control (16).

The majority of equine studies to date have concentrated on the pharmacokinetic profile of CBD. Reported oral dosing regimens in horses range from 0.1 to 3 mg/kg and are associated with plasma concentrations that rarely exceed 20 ng/mL (1, 18–28). Current data indicate that further research is necessary to clarify CBD pharmacokinetics and establish safe and effective dosing regimens and plasma targets in horses and ponies.

This study aimed to evaluate the effect of oral CBD oil administration (2 mg/kg BID over 21 days) on selected metabolic parameters, and to characterize the pharmacokinetics of CBD and its two main metabolites (7-OH-CBD and 7-COOH-CBD) in Connemara ponies. The main hypotheses were that this dosing regimen would 1) improve insulin and glucose responses during an oral sugar test (OST), reduce blood triglycerides, body weight, body condition score (BCS), and cresty neck score (CNS), and 2) produce measurable plasma concentrations of CBD and its primary metabolites. To the authors’ knowledge, this is the first study to investigate both metabolic effects and pharmacokinetics of CBD in ponies following 21 days of administration. The findings of the present study may provide a foundation for future pharmacodynamic investigations.

## Materials and methods

### Animals

Thirteen Connemara ponies (9 non-pregnant mares and 4 geldings; body weight 413–558 kg; height 145–154 cm; age 5–25 years) were enrolled in the study. Seven of the thirteen ponies were diagnosed with EMS based on the results of an OST.

All ponies were housed at the same facility, fed grass hay from hay nets, and had ad libitum access to water and salt. During the night, the animals were kept in individual boxes with straw bedding, and during the day they had access to pasture for approximately 8 h. Housing and dietary conditions remained unchanged throughout the experiment and for at least 6 weeks prior to its initiation. The trial was conducted between January and February.

No clinical signs were observed in any of the ponies before or during the experiment, and none had received any medications for at least 6 weeks prior to study initiation.

### CBD product

A commercially available CBD-containing hemp seed oil formulation (Hemp Arsenal®, THC-free CBD oil for horses; 20,000 mg CBD/100 mL) was used. The cannabinoid content of the product was independently verified by the CANNA Foundation laboratory.

### Experimental design and treatment protocol

Animals were randomly allocated to either a CBD-treated group (*n* = 7) or a control group (*n* = 6) using a stratified randomization approach. Stratification was performed based on two predefined variables: EMS status (EMS vs. non-EMS) and age (≤ 6 years vs. > 6 years), in order to ensure balanced distribution of these factors between groups. As a result, the CBD-treated and control groups included four and three ponies diagnosed with EMS, respectively. With regard to age, both groups comprised four animals older than six years. With respect to sex, the treated group included six mares, whereas the control group included three mares.

Horses assigned to the CBD group received an oral hemp seed oil formulation containing cannabidiol at a dose of 2 mg/kg body weight, administered twice daily for 21 consecutive days. The control group received an equivalent volume of flaxseed oil administered according to the same dosing schedule.

The oral CBD dose was selected based on previously published studies in horses. Reported oral doses in equine studies most commonly range from 0.1 to 3 mg/kg, and CBD has been shown to be detectable in equine plasma even at lower doses (19, 20, 25–29). In addition, the same oral dose was used in the authors’ previous study (30). Based on this evidence, a dose of 2 mg/kg was selected for the present study.

CBD and flaxseed oil were administered orally [approximately 4–5 mL, depending on body weight)] using a syringe inserted into the oral cavity at the interdental space, between the incisors and premolars, in accordance with routine veterinary practice for per os drug administration in horses. During the experimental period, morning administration was followed by hay feeding from a hay net approximately 20 min after dosing. Prior to the morning administration, horses had no access to hay for approximately 10–12 h; however, as they were housed on straw bedding, complete fasting cannot be assumed due to voluntary straw consumption. Feeding management and intake conditions are known to influence behavioural and physiological responses in equids, particularly under controlled experimental settings (31).

Evening administration was performed in the fed state, as horses had access to hay from hay nets during the day and again in their stalls until approximately 19:00, ensuring a full gastrointestinal fill at the time of dosing.

### Physical examination, body weight, BCS and CNS

On the day preceding the start of the trial, a physical examination was performed in all horses. Heart rate, respiratory rate, and rectal temperature were recorded. Gastrointestinal motility was assessed in four abdominal quadrants and scored as absent (0), physiological (1), slightly increased (2), or hypermotile (3). Responsiveness to auditory stimuli (hand clap at 60 cm from the head) and the degree of ataxia were evaluated using numerical scoring systems. Ataxia was assessed using a 4-point scale: no change from the normal, non-sedated state (0); stable but swaying (1); swaying with leaning against the wall (2); and swaying with leaning against the wall accompanied by flexion of the carpal joints and/or crossing of the hind limbs (3). Responses to auditory stimuli were also scored on a 4-point scale: no reaction (0); slight head movement without limb movement (1); vigorous head movement without limb movement (2); and vigorous head movement with movement of one or more limbs (3) (32). This physical examination protocol was repeated on days 1 and 21. Body weight was calculated (33), and BCS (34) and CNS (35) were assessed on the first day and after the final day of treatment.

The horses were closely monitored throughout the experiment for adverse effects, including bradycardia or tachycardia, depression, hypersalivation, hypothermia, hyperesthesia, diarrhea, colic, urinary incontinence, mydriasis, incoordination, ataxia, and seizures (36, 37).

### OST and insulin, glucose and adrenocorticotropic hormone (ACTH) sampling

An OST was performed in all horses prior to the start of the trial to assess the insulin response and changes in blood glucose concentration. Following collection of a baseline blood sample for ACTH analysis in elder individuals (*n* = 8), corn syrup was administered orally at a dose of 0.45 mL/kg body weight (Karo Light Corn Syrup; ACH Food Companies, Inc.). Additional blood samples were collected at 60 and 90 min after administration for glucose and insulin analysis. The cut-off value for EMS was >63 μIU/mL, according to the ECEIM consensus statement (16). This procedure was repeated at the end of the treatment period.

Blood samples were collected from the jugular vein into serum separator tubes containing a clot activator (VACUETTE® Tube, 3.5 mL, CAT Serum Separator Clot Activator; Greiner Bio-One GmbH, Kremsmünster, Austria) for insulin and glucose assessment and into K3-EDTA tubes (Sarstedt, Nümbrecht, Germany) for ACTH analysis. Samples were maintained at room temperature for approximately 30 min prior to centrifugation at 3,200 rpm for 10 min. The resulting serum and plasma were then transferred to the laboratory for analysis.

Plasma insulin and ACTH concentrations were measured by a commercial laboratory (PraxisLab Ltd., Budapest, Hungary) using chemiluminescent immunoassays on Siemens Immulite 2000 analyzer. Blood glucose concentrations were determined at the same laboratory using a Beckman Coulter DxC 700 AU analyzer (Beckman Coulter Inc., Brea, CA, United States) with manufacturer-provided reagents.

### Blood hematology and biochemistry sampling

Blood samples were collected from the jugular vein into K3-EDTA tubes (Sarstedt, Nümbrecht, Germany) for complete blood count (CBC) analysis and into plain serum tubes (Sarstedt, Nümbrecht, Germany) for biochemical profiling, prior to the first and after the final oil administration. The biochemical panel included total protein, albumin, aspartate aminotransferase (AST), alanine aminotransferase (ALT), alkaline phosphatase (ALP), glutamate dehydrogenase (GLDH), total bilirubin, direct bilirubin, gamma-glutamyl transferase (GGT), creatine kinase (CK), lactate dehydrogenase (LDH), triglycerides (TG), glucose, creatinine, urea, sodium (Na), potassium (K), sodium-to-potassium ratio, chloride (Cl), calcium (Ca), magnesium (Mg), phosphate (P), iron (Fe), total cholesterol, and fructosamine.

Samples were analyzed by a commercial laboratory (Vet-Med-Labor Ltd., Budapest, Hungary). Hematological parameters were measured using an automated hematology analyzer (Advia 120 Hematology System, Siemens Healthineers, Erlangen, Germany). Serum biochemical analytes were determined using standard automated enzymatic and colorimetric assays on a clinical chemistry analyzer (Advia 1800 Clinical Chemistry System, Siemens Healthcare Diagnostics, Tarrytown, NY, United States), in accordance with the manufacturers’ instructions. Electrolyte concentrations were measured using ion-selective electrodes.

### Blood sampling for CBD and metabolites analyses

On day 1, following CBD administration, blood samples were collected at 2, 4, and 12 h. Additional samples were obtained at 48, 96, 144, and 192 h after the start of the experiment. On day 21 to evaluate potential alterations in pharmacokinetics following long-term administration, blood sampling was conducted according to the same schedule as described for day 1. To assess the elimination of cannabidiol and its metabolites, additional blood samples were collected from horses in the CBD-treated group on days 3, 7, 14, 21, 28, and 35 following the final administration.

Blood samples (9 mL) were collected via direct venipuncture using a 22-gauge needle into Vacuette® lithium heparin (LH) blood collection tubes (Greiner Bio-One GmbH, Kremsmünster, Austria). Samples were maintained at room temperature for approximately 30 min prior to centrifugation (3,200 rpm for 10 min). Plasma (4 mL) was then transferred into cryovials and stored at −20 °C until analysis.

### CBD and metabolites analysis in horse plasma

#### Sample preparation procedure

The liquid–liquid extraction procedure for pony plasma samples began by adding 10 μL of THC-D3 (100 ng/mL) internal standard solution, then 10 μL of methanol, followed by 400 μL of the plasma sample and 100 μL of saline solution. After 0.5 min vortexing, 1,000 μL of ethyl acetate was added for enrichment of CBD and its metabolites in the organic phase, then vortexed and sonicated for 5 min. For separating the organic and aqueous phases, 10 min centrifugation was used at 15000 rpm (Universal 320 R, Hettich, Tuttlingen, Germany) at 4 °C. The upper layer was collected into the microcentrifuge tube and evaporated to dryness. The dried extract was reconstituted in 100 μL methanol, and 7 μL aliquots were injected for UHPLC–MS/HRMS analysis. For the preparation of matrix-matched external calibration samples, the same extraction procedure described above was used, except that 10 μL of methanolic calibration mixture was added instead of 10 μL of methanol, resulting in 0, 1.25, 2.50, 12.5, 25.0, 50.0, 100 ng/mL in plasma for CBD and 7-OH-CBD, while this concentration range was extentended by the following member for 7-COOH-CBD: 200, 500, 1,000, 1,500, 2,000, 3,000 and 5,000 ng/mL.

### UHPLC–MS/HRMS for quantitation of CBD and its two main metabolites

The UHPLC–MS/HRMS analyses were performed on Waters Acquity I-Class UPLC (Milford, MA, UK) connected to Thermo Scientific Orbitrap Exploris 240 Plus Hybrid Quadrupole-Orbitrap™ (Waltham, MA, United States) mass spectrometer. The UPLC system was controlled by the MassLynx software (Milford, MA, UK). Data were acquired and evaluated with Xcalibur 4.4 software (Thermo Fisher Scientific, Waltham, MA, United States).

For the UHPLC separations of analytes, Kinetex™ XB-C18 column (50 × 2.1 mm, 2.6 μm) with SecurityGuard Ultra C18 cartridge (4 × 3 mm) was used from Phenomenex (Torrance, CA, United States). The UHPLC mobile phase A consisted of 1 v/v% formic acid aqueous solution, and mobile phase B was composed of MeOH with 1 v/v% formic acid. The gradient program was 0–0.20 min 20% B, 0.20–1.00 – 3.00 min 20–100—100% B, 3.00–3.10—3.95 min 100–20 –20% B. The flow rate gradient program was 0–2.00 min 0.3 mL/min, 2.00–2.01 – 3.90 –3.95 min 0.3–0.5—0.5–0.3 mL/min. The column temperature was maintained at 50 °C; the autosampler temperature was set to 5 °C.

For MS/HRMS detection of targeted compounds, the positive heated-electrospray ionization (HESI) was set up with a capillary and vaporizer temperature of 250 °C, S-Lens RF level 50, spray voltage 4.5 kV, sheath gas flow 50, auxiliary gas flow 10, and sweep gas flow 1 in arbitrary units. The scheduled parallel reaction monitoring parameters were as follows: a resolution of 15,000 (FWHM), RF 70 Lens (%), AGC setting of 1 × 10^5^ charges, and a maximum isolation time of 50 ms. The width of the isolation window of the precursor ion was 0.5 Da. The optimized collision energies were the following for the protonated form of CBD (22 eV), 7-OH-CBD (24 eV), 7-COOH-CBD (20 eV), and THC-D3 (22 eV). To prevent contamination of the mass spectrometer, the UHPLC effluent was directed to the HESI source only between 1.2 and 2.0 min using a six-port divert valve; during the remaining time, the system was flushed with 90/10 (v/v) acetonitrile/water using Waters Acquity I-Class auxiliary pump (Milford, MA, UK).

### Statistical analysis

All statistical analyses were conducted using R version 4.4.2 (R Developmental Core Team 2024). Due to variation in measured parameters across age and sex groups, individuals were categorized into two age groups [elder (> 6 years; *n* = 8) and young (≤ 6 years; *n* = 5)] and two sex groups (mares, *n* = 9; geldings, *n* = 4). To account for kinship among individuals, a family identifier (ID) based on mother–daughter relationships was included in the analyses as a six-level factor when necessary. One gelding was the sire of multiple individuals and was therefore assigned a unique family identifier.

A quantile-quantile plot statistical descriptive analysis was performed to assess data normality using the “qqnorm” and “qqline” functions (38). Data from physical examinations were compared using the heart rate, respiratory rate, rectal temperature, and gastrointestinal motility as dependent variables. Further tested parameters were the degree of ataxia, auditory response score, body weight, BCS, and CNS.

There was no variability among individuals in ataxia or gastrointestinal motility; therefore, these variables were not included in the statistical analyses.

Linear mixed models were fitted for heart rate, respiratory rate, and body temperature. Independent variables included time of measurement (three-level factor), sex, age (two-level factor), and treatment group (control or CBD). The interaction between time of measurement and treatment group was also tested. Individual ID and family ID were included as random effects (random intercepts). For modelling CNS variable, treatment group and time of measurement (two-level factor) were used included as independent variables. The pre-treatment time point was used as the reference level in all analyses.

To calculate the exact difference between measurements post-hoc comparisons (with Dunnett contrasts) were used, where all time were compared with the reference level. The auditory stimulation response scores, the median BCS values were compared with Wilcoxon test (pairwise comparison with the reference level) in each treatment group separately.

For comparing the blood hematology and biochemistry parameters before and after treatment linear mixed models were used. In each model, the independent variables included time of measurement (two-level factor), sex, age (two-level factor), and treatment group (control or CBD). The interaction between time of measurement and treatment group was also tested. Individual ID and family ID were included as random effects (random intercepts). To account for multiple comparisons, the Bonferroni correction was applied in all models.

Linear mixed models were used to analyze the results of the OST before and after CBD treatment, with blood glucose and insulin as dependent variables. For elder individuals, ACTH levels were also included as a dependent variable. In the blood glucose and insulin models, independent variables included time of measurement (two-level factor: before or after treatment), sex, age (two-level factor), treatment group (control or CBD), and time of blood sampling (two-level factor for blood glucose and insulin). Individual ID and family ID were included as random effects (random intercepts). Interactions between time of measurement and treatment group, time of blood sampling and treatment group, and time of measurement and time of blood sampling were tested.

For linear mixed models the *lmer* function [package “lme4”; (39)], for post-hoc comparisons the *contrast* function [package “emmeans,” method = Dunnett; (40)] were used. The level of significance for vital parameters, degree of ataxia, auditory response score, BCS, CNS and OST models were tested using analysis of variance with type-2 sums of squares tests [package “car”; (41)]. For blood hematology and biochemistry *glht* function with Bonferroni correction [package “multcomp”; (42)] were used. Model assumptions—including normality of residuals and random effects, linearity, homogeneity of variance, and multicollinearity—were visually assessed using the *check_model* function in R package “performance” (43). Visualizations of the results were created using the *ggplot* function (44).

### Pharmacokinetic analysis

Pharmacokinetic parameters for CBD its metabolites were calculated using non-compartmental analysis by Phoenix WinNonlin Software, version 8.5.2.4 (Certara Inc., Pennsylvania, United States). The maximum observed plasma concentration (C_max_) and the time of maximum observed plasma concentration (*t*_max_) were determined based on the plasma concentration-time data. The terminal elimination rate constant (λ_z_) was estimated by linear regression of the terminal portion of the log concentration-time curve. Terminal elimination half-life (t_½_) was calculated as *t*_½_ = ln2/ λ_z_. The apparent volume of distribution based on the terminal elimination phase (V_z_/F) and the apparent oral clearance of drug from plasma (Cl/F) were calculated using the formula of Dose/(λ_z_·AUC_0-*τ*_) and Dose/A AUC_0-τ_, respectively, where F is the fraction of dose absorbed. The area under the plasma drug concentration-time curve from dosing time to dosing time plus 12 h (AUC_0-τ_) was determined by linear trapezoid rule. The area under the plasma drug concentration-time curve from time 0 to infinity (AUC_0-∞_) and the area under the plasma drug concentration-time curve from the time of the last dose to infinity (AUC_504-∞_) were estimated as AUC_last_ + C_last_/λ_z_. The percentage of AUC_0-∞ or 504-∞_ due to extrapolation from the last measurable concentration to infinity was calculated with the formula 100·(AUC_0-∞ or 504-∞_—AUC_last_)/ AUC_0-∞ or 504-∞_). The mean residence time from time 0 to infinity (MRT_0-∞_) was estimated with the formula of MRT_0-∞_ = AUMC_0-∞/_AUC_0-∞_, where AUMC_0 inf_ is the area under the first moment curve extrapolated to infinity. The accumulation ratio (R_ac_) indicates ratio of accumulation of a drug in the body after multiple doses, compared to a single dose. R_ac_ was calculated as AUC_0 *τ* (Last dose)_/AUC_0-τ (First dose)_. The steady state was considered to be reached when at least two consecutive pre-dose concentrations differed by ≤10% and remained within the method’s analytical CV.

## Results

### Blood insulin and glucose responses during the OST

**Figure 1:**
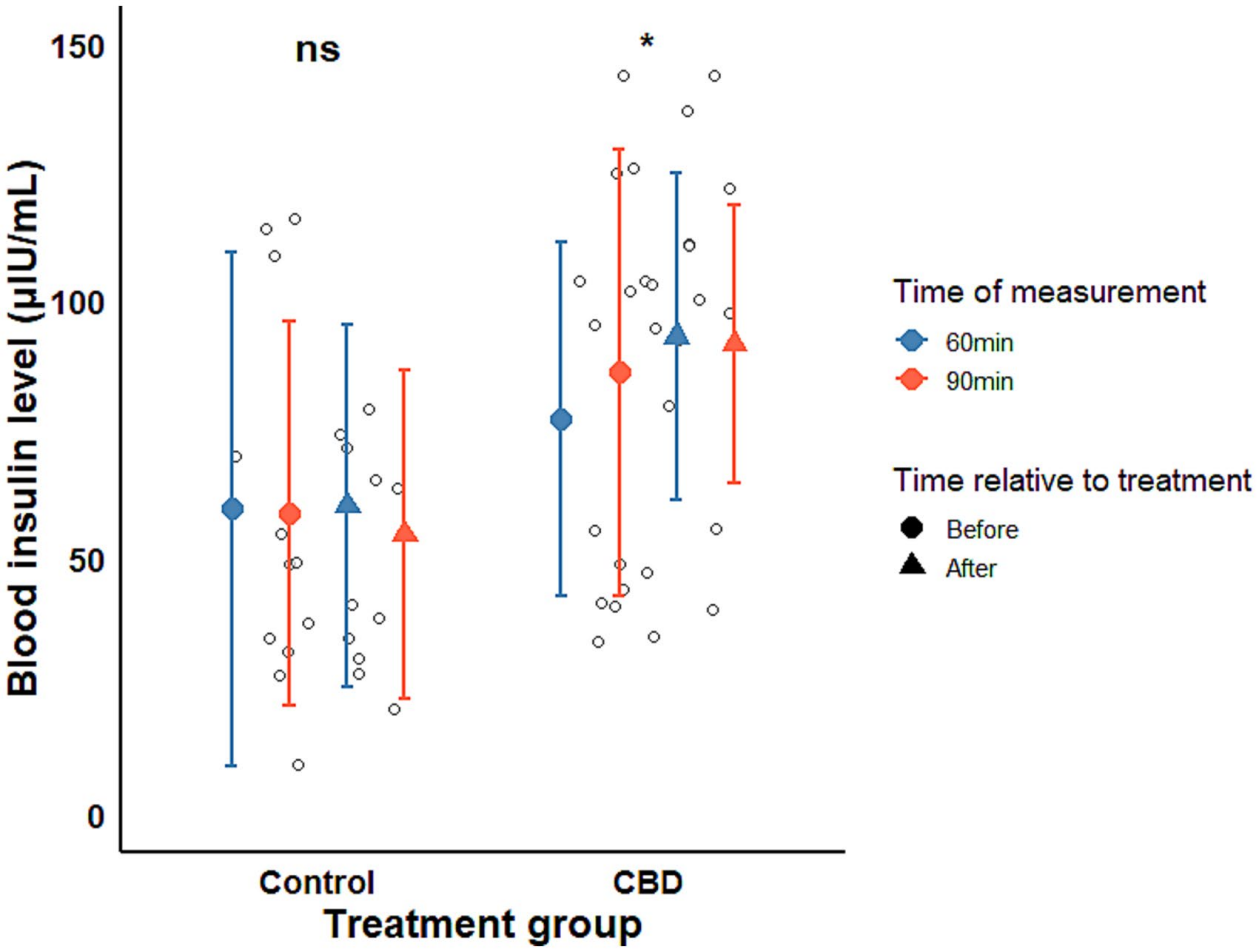
Blood insulin concentrations during the OST by time of measurement and sampling time. Means and confidence intervals (CI) were calculated from the raw data. Significant differences are indicated by asterisks; *p*-values were obtained from *post hoc* comparisons of the linear mixed-effects model.

Prior to CBD administration, mean insulin concentrations during the OST were 59.40 and 77.01 μIU/mL at 60 min, and 58.62 and 86.04 μIU/mL at 90 min in the control and CBD-treated groups, respectively, with higher values observed in the CBD-treated group at both time points. Following the treatment period, insulin concentrations remained numerically higher in the CBD-treated group at 60 and 90 min (60.07 vs. 92.97 μIU/mL at 60 min; 54.45 vs. 91.57 μIU/mL at 90 min; Figure 1). Given the baseline differences between groups, the primary outcome of interest was the within-group change in insulin concentrations over time rather than absolute between-group comparisons. A significant increase in mean insulin concentrations during the OST was observed in the CBD-treated group following the treatment period (estimate ± SE = 10.74 ± 5.19 μIU/mL; *t*-ratio = 2.072, *p*-value = 0.046), whereas no significant change was detected in the control group (estimate±SE = −6.21 ± 6.14 μIU/mL, *t*-ratio = −1.012, *p*-value = 0.319). This increase in insulin response may be of potential clinical relevance in EMS-affected animals, as exaggerated insulin responses are associated with laminitis risk; however, the functional significance of this finding remains uncertain and requires further contextual interpretation.

**Figure 2:**
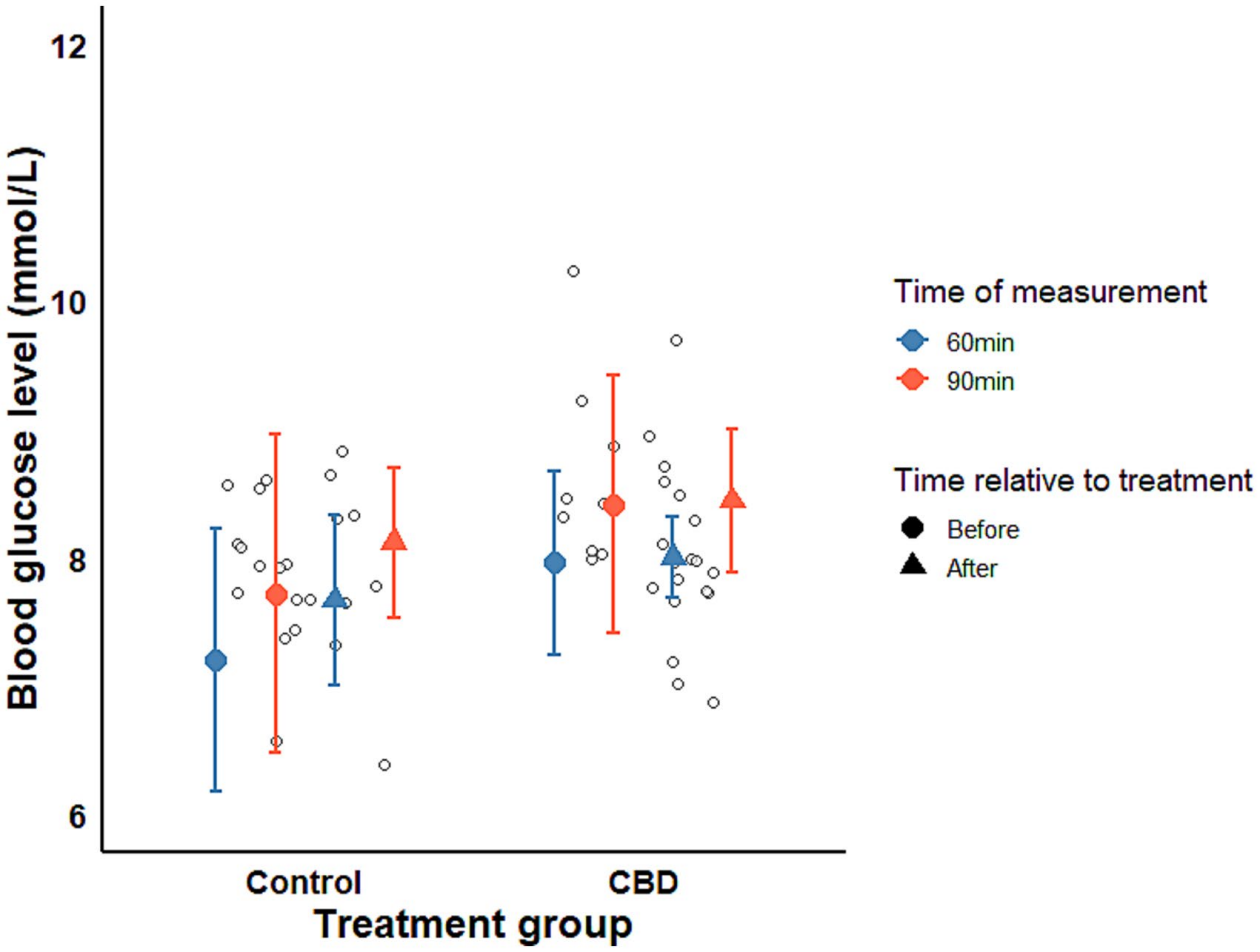
Blood glucose concentrations during OST by time of measurement and sampling time. Means and CIs were calculated from the raw data.

Mean blood glucose concentrations did not differ significantly between treatment groups (*χ^2^* = 0.653, *p*-value = 0.419) or between pre and post-treatment measurements (*χ^2^* = 0.815, *p*-value = 0.366). Absolute glucose values are provided in Supplementary Table S2. A significant increase was observed only between 60 and 90 min, consistent with the expected post-treatment glycemic response (Figure 2).

### Physical examination, body weight, BCS and CNS

The product was easily administered orally using a dosing syringe and was well tolerated by all horses. No adverse effects were observed throughout the study. None of the horses showed any signs of discomfort or reduced appetite. Body temperature, heart rate, and respiratory rate remained within normal physiological ranges for all 13 ponies.

**Figure 3:**
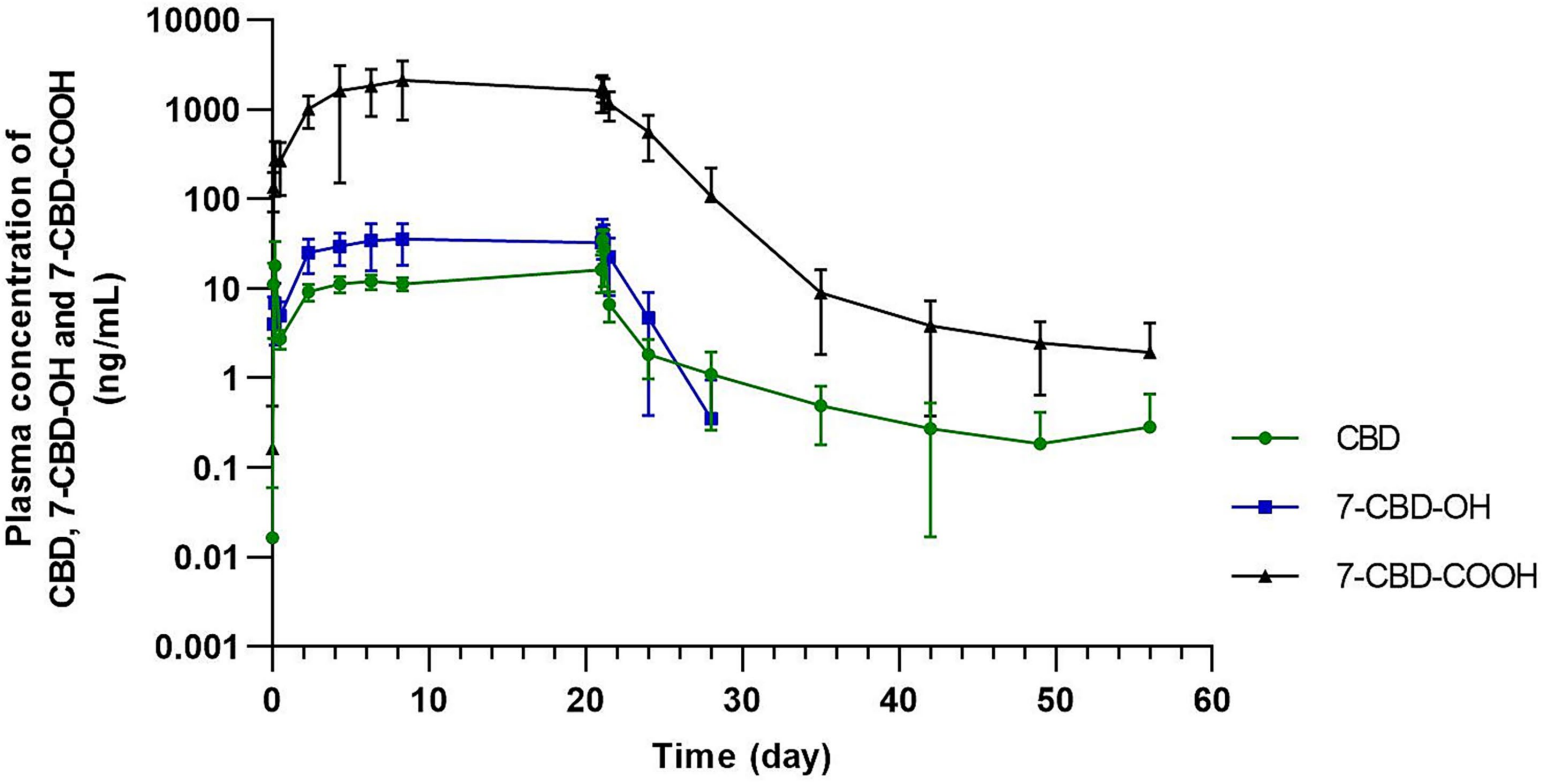
Mean ± standard deviation plasma concentration–time profiles of CBD and its metabolites on a semi-logarithmic scale over the entire study period, including the accumulation phase during repeated dosing and the terminal elimination phase after the final dose of CBD (2 mg/kg every 12 h for 21 days).

Mean heart rate was significantly higher in the CBD group (*χ^2^* = 12.203, *p* < 0.001), with a significant sex × time effect (*χ^2^* = 10.707, *p* = 0.004). In mares, no significant changes were observed across time points. In geldings, mean heart rate decreased compared to pre-treatment values (24 h post-treatment: estimate ± SE = −5.33 ± 1.88, *t* = −2.844, *p* = 0.022; day 21: −6.67 ± 1.88, *t* = −3.556, *p* = 0.005; Figure 3). No significant interaction was found between treatment group and examination time points. Mean body mass did not change following treatment [before (mean±SE) = 477.77 ± 9.620 kg, after = 484.08 ± 9.877 kg; *χ^2^* = 2.337, *p* = 0.126] and did not differ between treatment groups (control = 470.667 ± 8.925 kg, CBD = 489.71 ± 9.680 kg; *χ^2^* = 0.526, *p* = 0.468).

The median BCS did not change significantly within either group during the treatment period {before [median(IQR)] = 6.0 (5.0–7.0), after = 6.0 (6.0–6.0)}. Although median BCS values were numerically higher in the CBD-treated group [control = 5.5 (5.0–6.0), CBD = 7.0 (6.0–7.0); W = 139, *p* = 0.003].

The CNS value did not differ between the treatment groups [control(mean±SE) = 2.084 ± 0.084, CBD = 2.71 ± 0.221], only marginally decreasing after treatment process was found (before(mean±SE) = 2.54 ± 0.183, after = 2.31 ± 0.208; estimate±SE = −0.250 ± 0.131, *t*-value = 1.915, *p*-value = 0.055).

No significant differences were detected in respiratory rate, body temperature, or response scores to auditory stimulation across the three physical examination time points or between the two treatment groups.

### Blood hematology, biochemistry and ACTH level

Blood hematology and biochemistry analyses were performed at baseline and at the end of the 21-day trial period. The measured average values are provided in the table attached as (Supplementary Table S1).

At baseline, the WBC count (reference range: 5–10 G/L) was within normal limits in all subjects. However, by the end of the trial period, a reduction was observed in four ponies (4.6–4.9 G/L), two of which belonged to the treatment group. The HCT level (reference range: 32–42%) was below the normal range in one pony at all time points (31 and 28%), although this deviation was considered mild.

The ALT level (reference range: 1–15 IU/L) was within the normal range at baseline for all subjects. However, by the end of the trial, ALT levels were elevated in all horses, regardless of treatment. In two horses (one from the CBD group and one from the control group), ALT levels were slightly above the normal range (16 IU/L). The ALP level (reference range: 147–261 IU/L) was elevated at baseline in five ponies (276–334 IU/L). However, by the end of the trial, only one pony showed a slight elevation (281 IU/L). Three of the five ponies with elevated ALP levels were in the CBD group.

At baseline, resting blood glucose levels (reference range: 3–5 mmol/L) were elevated in four ponies (5.2–5.5 mmol/L), all of which were diagnosed with EMS, and two of them were in the CBD treatment group. Regardless of treatment, 11 ponies had elevated blood glucose values (5.1–5.8 mmol/L) by the end of the trial, including one additional pony with EMS and six non-EMS ponies. Six of these ponies received CBD treatment. Fructosamine levels (reference range: 120–290 μmol/L) were elevated in 9 out of 13 subjects (301–338 μmol/L), six of which were diagnosed with EMS. However, regardless of treatment, all ponies had normal fructosamine levels by the end of the trial. TG levels (reference range: 0.02–0.5 mmol/L) exceeded the normal range in eight ponies (0.52–0.69 mmol/L), five of which had EMS. By the end of the trial, 11 ponies had elevated TG levels (0.58–0.91 mmol/L), seven of which received CBD treatment. The mean TG level increased in the CBD treatment group, from 0.6 to 0.74 mmol/L, while in the control group, it remained relatively stable, changing from 0.51 to 0.5 mmol/L.

Urea levels (reference range: 3.3–6.7 mmol/L) were slightly reduced in two horses (3.0 and 3.2 mmol/L); however, these levels returned to normal at the second sampling. Iron levels (reference range: 14.5–25 μmol/L) were elevated in twelve ponies (26.5–41.5 μmol/L) at baseline, and in eleven ponies (25.4–59.5 μmol/L) at the conclusion of the trial. CK levels (reference range: 60–330 IU/L) were within normal limits at baseline. However, two horses (340 and 368 IU/L) slightly exceeded the normal range at the second sampling. LDH levels (reference range: 225–700 IU/L) were elevated in nine horses (707–1,268 IU/L) at baseline, and in ten horses (772–1,372 IU/L) by the end of the trial. Although total bilirubin concentrations remained within the reference range (7–60 μmol/L) at all time points in all ponies, the CBD-treated group showed significantly lower baseline values compared with controls (estimate ± SE = 5.668 ± 1.863 μmol/L, *z* = 3.043, *p* = 0.012). Total bilirubin concentrations increased after treatment independently from the treatment group (estimate ± SE = 5.233 ± 1.571 μmol/L, z = 3.332, *p* = 0.004), while remaining within normal limits.

Apart from total bilirubin, none of the measured parameters differed significantly between the control and treatment groups, nor were they directly influenced by CBD treatment.

For assessment of pituitary pars intermedia dysfunction (PPID), the commercial laboratory reference interval defined ACTH concentrations < 15 pg./mL as normal, whereas values > 40 pg./mL were considered indicative of PPID. At the initial ACTH evaluation, only 2 of the 8 elder ponies showed a mild elevation in ACTH concentrations (17.1 and 18.4 pg./mL). After the final dose, elevated ACTH concentrations were observed in 5 ponies, ranging from 15.8 to 21.5 pg./mL.

In both the control and CBD-treated groups, mean ACTH concentrations increased significantly by the final day of treatment, from 14.03 to 17.68 pg./mL and from 11.93 to 16.48 pg./mL, respectively (estimate ± SE = 4.086 ± 0.731 pg./mL, *t* = 5.589, *p* < 0.001). This increase was observed in all 13 horses, irrespective of treatment group.

### Pharmacokinetics of CBD and its main metabolites

**Figure 4:**
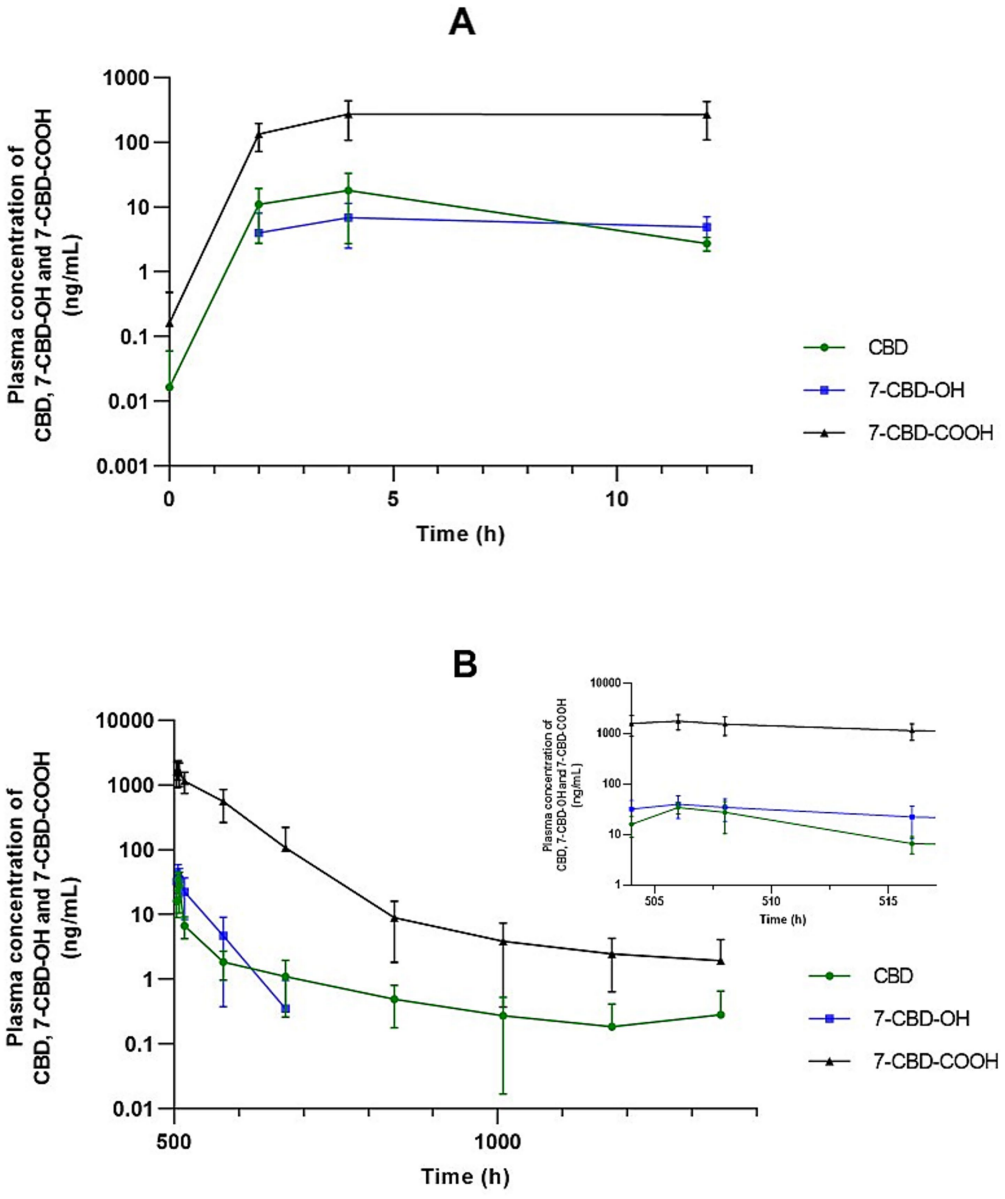
Mean ± standard deviation plasma concentration–time profiles of CBD and its metabolites on a semi-logarithmic scale. **(A)** Following the first dose. **(B)** Following the last dose. The inset shows the terminal phase after the final administration.

The estimated pharmacokinetic parameters for CBD and its metabolites after the first and last doses following oral administration of CBD at 2 mg/kg every 12 h are summarized in Table 1. After administration of the first dose of CBD, the C_max_ of CBD was 22.79 ± 13.24 ng/mL, whereas by the end of the study, following the final dose, it had increased to 39.93 ± 14.45 ng/mL. The t_max_ was 3.14 ± 1.07 h after the first dose and 2.29 ± 0.76 h after the last dose, while the t_½_ (elim) was 5.48 ± 2.06 h and 4.41 ± 1.23 h, respectively. With an accumulation ratio of 2.75 ± 1.51, CBD demonstrated moderate accumulation in the subjects during repeated dosing. Pre-dose concentrations increased markedly between 56 and 104 h (+23.5%), indicating that steady state had not yet been reached. In contrast, the changes between 104–152 h (+5.8%) and 152–200 h (−5.3%) were minimal, indicating stable concentrations. Therefore, steady state was confirmed at approximately 152 h, although concentrations at 104 h already approached the steady-state level. The overall concentration–time profiles over the entire study period are shown in Figure 3, while the concentration–time profiles following the first and last doses are presented in Figure 4.

**Table 1:** Mean ± standard deviation of estimated pharmacokinetic parameters for CBD and metabolites after the first and last dose following oral administration of CBD at 2 mg/kg every 12 h.

Parameter	Dosing regimen	
	After first dose (*n* = 7)	After last dose (*n* = 7)
CBD		
C_max_ (ng/mL)	22.79 ± 13.24	39.93 ± 14.45^*^
t_max_ (h)	3.14 ± 1.07	2.29 ± 0.76 ^ns^
t_½ (elim)_ (h)	5.48 ± 2.06	4.41 ± 1.23 ^ns^
λ_z_ (1/h)	0.14 ± 0.05	0.18 ± 0.08 ^ns^
V_z_/F (mL/kg)	156350.50 ± 117916.16	59966.30 ± 23267.52 ^ns^
Cl/F (mL/kg/h)	18384.77 ± 7379.70	9180.80 ± 1565.62^**^
AUC_0-*τ*_ (h*ng/mL)	101.15 ± 47.97	223.12 ± 36.61^***^
AUC_0-∞_ (h*ng/mL)	123.37 ± 44.40	—
AUC_504-∞_ (h*ng/mL)	—	269.01 ± 31.29
AUC_0-∞ or 504-∞_ extrapolation (%)	21.02 ± 14.42	17.22 ± 8.03 ^ns^
MRT_0-∞_ (h)	8.68 ± 3.20	7.29 ± 1.58 ^ns^
R_ac_	—	2.75 ± 1.51
7-OH-CBD		
C_max_ (ng/mL)	8.38 ± 4.60	40.17 ± 19.01 ^**^
t_max_ (h)	6.00 ± 4.16	2.00 ^*^
AUC_0-τ_ (h*ng/mL)	60.51 ± 25.97	368.60 ± 163.58 ^***^
Ratio: AUC_0-τ_ (CBD-OH)/AUC_0-τ_ (CBD)	0.61 ± 0.08	1.69 ± 0.86 ^**^
7-COOH-CBD		
C_max_ (ng/mL)	232.53 ± 80.00	1611.58 ± 395.89^***^
t_max_ (h)	8.00 ± 4.38	2.00^**^
AUC_0-τ_ (h*ng/mL)	2207.19 ± 735.99	15535.67 ± 3774.95^***^
Ratio: AUC_0-τ_ (7-COOH-CBD)/AUC_0-τ_ (CBD)	27.78 ± 10.72	71.77 ± 22.83^***^

C_max,_ maximum plasma concentration; t_max_, time of maximum observed plasma concentration; t_½_, terminal elimination half-life; λ_z_: terminal elimination rate constant; V_z_/F, apparent volume of distribution based on the terminal elimination phase; Cl/F, apparent oral clearance of drug from plasma; AUC_0-τ_, area under the plasma drug concentration-time curve from dosing time to dosing time plus 12 h; AUC_0-∞_, area under the plasma drug concentration-time curve from time 0 to infinity; AUC_504-∞_, area under the plasma drug concentration-time curve from the time of the last dose to infinity; AUC_0-∞ or 504-∞_ extrapolation (%), percentage of AUC_0-∞ or 504-∞_ due to extrapolation from the last measurable concentration to infinity; MRT_0-∞_, mean residence time from time 0 to infinity; R_ac_, accumulation ratio calculated as AUC_0-τ (Last dose)_/AUC_0-τ (First dose)_.

^ns^*p* > 0.05; ^*^p < 0.05; ^**^ *p* < 0.01; ^***^ *p* < 0.001 relative to the parameter observed after the first dose.

No cannabinoids were detected in the control plasma samples. In five of the seven treated horses, CBD was still detectable on day 35 after administration of the final dose, whereas in the remaining two it was measurable on day 14 but had fallen below the quantitation limit (0.100 ng/mL) by day 21. The 7-OH-CBD metabolite was below the detection limit in 1 horse by day 3, in 4 horses by day 7, and in 2 horses by day 14 following administration of the final CBD dose. However, the 7-COOH-CBD metabolite remained detectable in all subjects even 35 days after the final dose.

## Discussion

In the present study, oral administration of CBD at 2 mg/kg twice daily for 21 days did not significantly affect body weight, BCS, CNS, or blood triglyceride concentrations, but was associated with an increased insulin response during the OST.

EMS, which shares pathophysiological similarities with MetS, is characterized by insulin dysregulation, impaired glucose tolerance, insulin resistance, and dyslipidaemia, including hypertriglyceridaemia (16). Similarly, MetS is a multifactorial and modifiable condition associated with increased risk of cardiovascular disease, type 2 diabetes, and other adverse health outcomes, driven by mechanisms such as insulin resistance, adipose tissue dysfunction, systemic inflammation, and dyslipidaemia (45).

Preclinical studies indicate that CBD exerts beneficial effects on body weight and food intake regulation, glucose and lipid metabolism, insulin resistance, neuroinflammation, and obesity-related behavioural disturbances (46–54). Although the mechanisms of CBD are not yet fully understood, several central and peripheral targets have been proposed based on preclinical and *in vitro* studies in the context of obesity and metabolic syndrome. For example, CBD has been suggested to interact with cannabinoid (CB1, CB2) and serotonin (5-HT1A) receptors, which may contribute to effects on appetite regulation and reward-related feeding behaviour (46). Peripherally, CBD may improve insulin sensitivity and lipid metabolism through cannabinoid receptor modulation and activation of peroxisome proliferator-activated receptor gamma (PPARγ), potentially promoting adipose tissue browning, enhancing glucose utilization, and reducing dyslipidaemia. Additionally, CBD has been reported to exert anti-inflammatory effects and may lower circulating lipid levels via regulation of low-density lipoprotein metabolism. Together, these interconnected mechanisms may support the therapeutic potential of cannabidiol in managing obesity and related metabolic disturbances (46).

In the current study, body weight and BCS remained stable during the treatment period, and the observed difference in BCS between groups is likely attributable to baseline variability rather than a treatment effect. CNS scores showed only a marginal decrease following treatment. In contrast, in a mouse model of high-fat diet–induced obesity, longer-term CBD administration at a higher dose (25 mg/kg for 9 weeks) reduced weight gain, lowered serum glucose, and decreased white adipose tissue mass and adipocyte size (55). In the ponies included in this trial, TG levels remained stable in controls and showed a non-significant increase in treated ponies. Blood glucose concentrations during OST largely followed the expected post-prandial pattern in all groups. Insulin responses during the OST increased in CBD-treated ponies, while responses in control animals remained stable. The observed increase following CBD administration should be interpreted within the context of a longitudinal, within-subject effect. Although baseline insulin levels were higher in the CBD-treated group, the stability of insulin responses in control animals across both OSTs supports a treatment-associated change rather than random variability. Nevertheless, residual baseline differences cannot be entirely excluded, and confirmation in independent cohorts is warranted.

Diet composition is known to significantly influence metabolic responses and gut homeostasis in horses, with starch-rich conditions promoting dysbiosis and inflammatory pathways that may affect glucose–insulin dynamics (56). More generally, nutritional factors, particularly forage quality and dietary composition, play a crucial role in modulating metabolic health and digestive function in horses (57), while feeding behaviour and time-activity patterns are closely linked to metabolic regulation and overall physiological balance (58). Importantly, in the present study, feeding conditions and diet composition were consistent across all animals; however, differences in insulin response were observed only in the CBD-treated group, suggesting that the observed effect is unlikely to be attributable to dietary variation alone. Nevertheless, the study design does not allow differentiation between a potential worsening of insulin dysregulation and a modulation of insulin secretion dynamics, and therefore this finding should be interpreted with caution.

The observed effects of CBD may vary depending on multiple contributing factors, including species-specific receptor distribution and the targeted clinical outcome. Cannabinoid receptor localization and ligand binding differ across species, indicating that pharmacological responses may not be directly comparable (59, 60). In addition, CBD exerts dose-dependent effects via multiple cellular targets, with distinct plasma concentrations required for different clinical outcomes; such actions, mediated by receptor activation, antagonism, or inhibition, have been previously described (61).

Rafailovska et al. (62) demonstrated that the metabolic effects of CBD are both dose and route-dependent. In healthy rats, intragastric CBD improved glucose regulation only at the highest dose tested (50 mg/kg), whereas lower doses (0.5 and 5 mg/kg) were ineffective, and intraperitoneal administration produced no significant glycemic effects. In diabetic rats treated intragastrically for 8 days, CBD exerted dose-specific metabolic effects: 25 mg/kg reduced blood glucose and increased insulin levels, while the most pronounced metabolic improvements were observed at 50 mg/kg, including the greatest reduction in glucose concentrations, a moderate rise in insulin, suppression of gluconeogenic enzymes, and improvements in lipid parameters. In contrast, the highest dose (100 mg/kg) showed minimal metabolic benefit. Overall, these findings emphasize the critical role of both dose and administration route in determining CBD’s metabolic efficacy.

It is possible that higher doses of cannabidiol attenuate its effects on glycaemic control through receptor desensitization, activation of counter-regulatory mechanisms, or non-specific interactions with additional molecular targets that influence metabolic pathways (62).

While increases in insulin concentrations could raise concerns in EMS-affected horses, particularly with respect to laminitis risk, existing experimental evidence indicates that CBD may improve metabolic parameters at certain doses, even in the presence of moderate insulin elevation. This suggests that the metabolic effects of CBD may depend on a balance between dose, metabolic status, and species, highlighting the need for further targeted studies. From a clinical perspective, these findings suggest that CBD use in EMS-prone horses or ponies should be approached with caution, particularly with regard to dose selection and metabolic status, until more robust evidence becomes available. Although CBD was well tolerated and did not adversely affect most metabolic parameters, the potential for enhanced post-prandial insulin response may be clinically relevant, given the established link between hyperinsulinemia and laminitis risk. Therefore, until further evidence is available, the use of CBD in EMS-prone individuals should be approached cautiously and under close veterinary supervision. In horses, positive clinical effects on mechanical allodynia and crib-biting have been reported at relatively low oral doses of cannabidiol (0.5 mg/kg twice daily), despite the typically low systemic cannabidiol concentrations observed at these doses in the literature (10, 11).

Collectively, these findings indicate that varying plasma cannabidiol concentrations can be clinically relevant and emphasize the need for dose-escalation studies with integrated pharmacokinetic analyses and efficacy assessments across multiple health conditions to more precisely define therapeutic exposure ranges. Future studies should also investigate longer-term administration protocols and dose–response relationships, with particular emphasis on insulin dynamics in EMS-affected horses, and consider the application of advanced monitoring approaches to better characterize metabolic responses under field conditions.

In the present study, oral CBD administration was well tolerated in all horses, with no adverse effects or changes in appetite observed throughout the study. Physiological parameters remained within normal ranges, indicating a favorable safety profile. Mean heart rate was within physiological limits in all animals. Although minor group and sex-related variations were observed, these differences were small in magnitude and are unlikely to be clinically relevant with respect to the primary metabolic outcomes of the study. These findings should nevertheless be interpreted with caution given the limited sample size.

Biochemical analyses revealed minor changes in ALT, glucose, fructosamine, total bilirubin, and triglycerides over the 21-day trial. ALT levels showed slight elevations in all horses, independent of treatment. Blood glucose and triglycerides were elevated in several ponies, particularly those with equine metabolic syndrome, with changes occurring regardless of CBD administration. Fructosamine levels, which were elevated in some ponies at baseline, normalized by the end of the trial and were not influenced by CBD treatment. Total bilirubin increased slightly in both groups but remained within normal limits, with no evidence of CBD-related effects. Overall, these findings indicate that chronic CBD administration did not adversely affect key biochemical parameters.

Evidence from other species highlights the potential for dose-dependent liver effects of CBD. In humans, therapeutic CBD (1,500 mg/day) caused ALT elevations in some healthy adults, and Ewing et al. (63) demonstrated dose-dependent liver injury in mice at doses above 50 mg/kg. In contrast, studies in horses suggest that modest, long-term oral CBD administration is well tolerated. Wang et al. (64) observed normal serum liver enzymes and unremarkable hepatic biopsies in horses treated with 1 mg/kg twice daily for 6 weeks. Similarly, Yocom et al. (28) reported transient, non-dose-dependent elevations in liver enzymes (GGT, AST, sorbitol dehydrogenase) in horses administered 0.5 or 1.5 mg/kg CBD orally twice daily for 6 weeks, which returned to reference intervals within 10 days. In the authors’ previous study, only slight, clinically irrelevant increases in GGT, total bilirubin, and LDH were observed (30). These results suggest that modest, long-term doses of these cannabinoids appear safe in horses, though further studies are needed to evaluate the effects of higher doses or prolonged administration on liver function and overall drug efficacy.

Beyond liver safety, ACTH concentrations increased in all eight elderly horses over the study period independent of treatment. A comparable pattern was observed for blood glucose, which increased in most ponies by the end of the trial regardless of treatment or EMS status. Previous studies have shown that even short-term stressors, such as road transport, can induce elevations in circulating ACTH and glucose concentrations in horses (65, 66). The generalized nature of these elevations, including in non-EMS animals, supports the interpretation that stress associated with the experimental conditions contributed to the observed changes rather than a direct metabolic effect of CBD.

The second aim of the present study was to characterize the plasma pharmacokinetics of CBD and its two main metabolites (7-OH-CBD and 7-COOH-CBD). Repeated oral administration of CBD at 2 mg/kg every 12 h resulted in an increase in peak plasma concentrations from the first to the final dose, consistent with the observed accumulation ratio (Table 1). Specifically, after administration of the first dose, the C_max_ of CBD was 22.789 ± 13.235 ng/mL, whereas by the end of the study, following the final dose, it had increased to 39.933 ± 14.448 ng/mL. Overall maximal plasma concentrations were consistent with those reported in the existing equine literature, while variability among studies likely reflects differences in dose, formulation, route of administration, and dosing regimen, as well as individual animal characteristics (3).

Oral administration has consistently been associated with dose-dependent increases in C_max_ (29). For example, Thomson et al. (67) reported peak plasma concentrations of approximately 40 ng/mL following administration of 8 mg/kg CBD via nasogastric tube, while Sánchez de Medina et al. (68) observed higher C_max_ values after oral administration of 10 mg/kg CBD, particularly when using oil-based and micellar formulations. The markedly higher plasma concentrations achieved with micellar preparations suggest that formulation plays a critical role in enhancing CBD bioavailability. In addition, alternative administration routes that bypass first-pass hepatic metabolism, such as transmucosal delivery, have been shown to yield measurable plasma concentrations even at low doses (32). Feeding status may further influence oral absorption, as demonstrated in humans; however, in horses, a crossover study comparing oil and paste formulations administered under fed and fasted conditions did not identify significant differences in pharmacokinetic parameters, although the paste formulation achieved peak plasma concentrations more rapidly, suggesting formulation-dependent differences in absorption kinetics (69). Repeated dosing has also been associated with higher peak concentrations, potentially reflecting drug accumulation and improved absorption over time, as reported by Williams et al. (29) following daily administration of full-spectrum CBD pellets.

Time to peak concentration remained relatively stable, and steady-state plasma levels were reached after approximately 4–5 days of dosing, as indicated by consistent pre-dose concentrations. These results demonstrate predictable pharmacokinetics and support the feasibility of twice-daily oral dosing in ponies.

Williams et al. (29) administered CBD orally to horses at 2.0 mg/kg once daily for 7 days. Plasma CBD concentrations increased over the initial dosing period and reached steady-state levels after approximately 3 days, with a mean C_max_ of approximately 51 ng/mL occurring at a T_max_ of about 2.4 h post-dose and a terminal half-life of about 10 h.

Sánchez de Medina et al. (68) conducted simulations of different CBD formulations and dosing regimens, using steady-state concentrations (after the 8th day) to calculate the probability of target attainment (PTA) for plasma levels ranging from 1 to 100 ng/mL. The simulations indicated that micellar formulations produced higher C_max_ and shorter t_max_, whereas oil formulations exhibited lower C_max_ but more stable concentrations at 24 h. Oral CBD at 10 mg/kg every 12 h was predicted to maintain plasma levels up to 40 ng/mL at steady state, while once-daily dosing achieved levels above 10 ng/mL throughout the dosing interval. However, these findings are based on simulations, and without clinical validation they remain predictive only.

In a multiple-dose study in horses, oral administration of CBD paste at 3 mg/kg twice daily for 15 days (*n* = 6) resulted in steady-state plasma concentrations being reached by Day 2 (20).

In a human multiple-dose study (*n* = 43), participants received an oil formulation at total daily CBD doses of 120, 240, 360, or 480 mg (with proportionally low THC content), administered orally in divided doses every 12 h for 7 consecutive days. Steady-state plasma CBD concentrations were achieved by Day 7, with higher CBD doses producing proportionally higher plasma CBD concentrations (70).

The t_max_ observed in the present study (Table 1) was consistent with most previous reports, which describe values ranging from 1 to 5 h (3).

In the present study, the t_½_ of CBD in horses was approximately 5.5 h after the first dose and 4 h after the final dose (Table 1). These values are shorter than those reported in most previous equine studies (10–12 h) (28, 29). In contrast, Eichler et al. (20) reported a markedly longer t_½_ of approximately 161 h following multiple-dose administration of CBD paste (3 mg/kg twice daily for 15 days). Conversely, Wang et al. (64) observed a substantially shorter elimination half-life, comparable to that reported in the present study, of approximately 4 h following oral CBD administration at 1 mg/kg twice daily for 6 weeks. In humans, the reported t_½_ was 2–5 days after chronic oral administration (28, 29, 71), while in dogs, *t*_½_ of orally administered cannabidiol vary widely across published pharmacokinetic studies, ranging from approximately 1 to 19 h (72).

Such variability in pharmacokinetic parameters across species, studies, and even individuals has been described following oral administration of cannabidiol formulations, even when doses are normalized. This variability may reflect small sample sizes, differences in sampling schedules, and interindividual factors such as breed, age, and sex. Age-related physiological changes affecting hepatic and renal function may further influence drug disposition, as may sex-related differences in metabolism. In addition, cannabidiol formulation and composition, whether purified or combined with other phytocannabinoids, may contribute to the observed pharmacokinetic variability (72).

Cannabinoid metabolism occurs primarily in the liver, where CBD is converted by cytochrome P450 enzymes to the active metabolite 7-OH-CBD and subsequently to the inactive metabolite 7-COOH-CBD (3). The latter persists longer in the body and is therefore considered a useful biomarker of cannabinoid exposure. CBD also undergoes direct glucuronidation via UDP-glucuronosyltransferases (73). Elimination occurs predominantly via feces, with a smaller proportion excreted in urine, mainly as unchanged or glucuronidated CBD (73, 74). Cannabinoid metabolic pathways vary considerably between species (25), and the specific metabolic profile of CBD in horses remains incompletely characterized. In humans, oral CBD undergoes extensive first-pass metabolism, resulting in rapid formation of metabolites (75, 76).

In the present study, repeated oral administration of CBD in ponies revealed a prolonged persistence of both the parent compound and its primary metabolite (7-COOH-CBD) in plasma following cessation of treatment. Cannabidiol remained detectable for up to 35 days after the final dose in most ponies, while the 7-COOH-CBD persisted in all animals through the final sampling time point. This extended detectability suggests slow elimination and possible metabolite accumulation in horses. These findings highlight the importance of considering extended metabolite persistence and elimination times when evaluating the safety, interpretation of plasma concentrations, and clinical use of CBD in equine practice.

Most international equine sport authorities prohibit all natural and synthetic cannabinoids; however, in 2022 the Fédération Equestre Internationale (FEI) reclassified CBD and its precursor cannabidiolic acid (CBDA) as controlled medications rather than prohibited substances (20). This regulatory shift highlights the need for a clear understanding of CBD pharmacokinetics in horses, as detection may still result in an adverse analytical finding but is evaluated under different rules that acknowledge potential inadvertent exposure.

The primary limitation of the present study was the small sample size (*n* = 13). Nevertheless, it should be noted that previous investigations addressing similar research questions have employed comparable sample sizes (9, 18, 19, 24, 25, 29, 30, 64, 68, 69). The consistency of the present results with those reported in the existing literature supports the adequacy of the sample size within this research context. Although stratified randomization was applied to balance key characteristics between groups, the relatively small sample size may have limited its effectiveness, and residual imbalance between groups cannot be excluded. In addition, it should also be acknowledged that clinical outcomes in equine studies are often influenced by multiple interacting physiological and environmental factors, reflecting the inherent biological variability of this species (77). Additional limitations of the present study include the exclusive analysis of blood samples, as urine samples were not collected; the relatively short treatment duration and fixed dosing regimen; and the lack of concurrent structured management interventions such as dietary restriction or exercise modification, which may also have influenced the observed outcomes.

## Conclusion

In conclusion, the hypothesis that CBD could reduce blood triglycerides, improve insulin and glucose responses, and decrease body weight, BCS, and CNS in Connemara ponies was not fully supported. The observed increase in insulin response during the OST suggests a CBD-associated modulation of insulin dynamics during a standardized oral sugar test, although the direction and physiological relevance of this effect in terms of insulin secretion versus insulin sensitivity cannot be fully determined.

While such increases in insulin concentrations could raise concerns in EMS-affected horses, particularly regarding laminitis risk, existing experimental evidence indicates that CBD may improve metabolic parameters at certain doses, even in the presence of moderate insulin elevation. This suggests that the metabolic effects of CBD likely depend on a balance between dose, metabolic status, and species, highlighting the need for further targeted studies. From a clinical perspective, these findings suggest that CBD use in EMS-prone horses or ponies should be approached with caution, particularly regarding dose selection and metabolic status, until more robust evidence becomes available.

Repeated oral administration was well tolerated, with no adverse effects on physiological or biochemical parameters. The hypothesis that CBD would produce predictable pharmacokinetics was confirmed, and steady-state levels were achieved within 4–5 days. Prolonged persistence of CBD and its metabolite 7-COOH-CBD highlights slow elimination and possible accumulation, emphasizing the need for further studies to optimize dosing and evaluate long-term safety and efficacy.

## Data Availability

The raw data supporting the conclusions of this article will be made available by the authors, without undue reservation.
