# Supplementary material for: Metabolic effects and pharmacokinetics of oral cannabidiol (CBD) in Connemara ponies following 21 days of treatment
Source: Front Vet Sci. 2026 May 14;13:1813917. doi: 10.3389/fvets.2026.1813917 (PMC13215832; doi:10.3389/fvets.2026.1813917)
Supplement: Supplementary file 1 [file Table_1.docx]

**Table S1:** Descriptive statistics of the measured haematological parameters in the Control (n= 6) and the CBD (n= 7) group 2 days before and 24 hours after treatment. In the case of every measured variable the mean, standard deviation (SD), median, standard error (SE), 95% confidence interval and range (differences between the highest and lowest value) were calculated from the original dataset. The calculation did not exclude any ponies.

|  |  | **Before treatment** | | | | **After treatment** | | | |
| --- | --- | --- | --- | --- | --- | --- | --- | --- | --- |
| **Measured variable** | **Reference level** | **Mean ± SD** | **Median ± SE** | **95% CI** | **Range** | **Mean ± SD** | **Median ± SE** | **95% CI** | **Range** |
| **Control group** |  |  |  |  |  |  |  |  |  |
| Alkaline phosphatase (ALP) | 147-261 IU/L | 241.83 ± 38.28 | 233.50 ± 15.63 | [211.20; 272.46] | 103.00 | 200.67 ± 32.63 | 199.00 ± 13.32 | [174.56; 226.78] | 88.00 |
| Glutamate dehydrogenase (GLDH) | 1-15 IU/L | 3.33 ± 2.16 | 3.00 ± 0.88 | [1.60; 5.06] | 6.00 | 4.00 ± 1.00 | 4.00 ± 0.58 | [2.87; 5.13] | 2.00 |
| Alanine aminotransferase (ALT) | 1-15 IU/L | 6.83 ± 1.33 | 7.00 ± 0.54 | [5.77; 7.90] | 4.00 | 14.00 ± 1.26 | 13.50 ± 0.52 | [12.99; 15.01] | 3.00 |
| Aspartate aminotransferase (AST) | 160-412 IU/L | 306.00 ± 36.38 | 311.00 ± 14.85 | [276.89; 335.11] | 101.00 | 315.17 ± 41.29 | 313.00 ± 16.86 | [282.13; 348.21] | 115.00 |
| Albumin | 26-41 g/L | 29.37 ± 1.89 | 29.65 ± 0.77 | [27.85; 30.88] | 4.40 | 32.37 ± 1.57 | 32.75 ± 0.64 | [31.11; 33.62] | 4.40 |
| Creatine kinase (CK) | 60-330 IU/L | 207.67 ± 20.18 | 209.50 ± 8.24 | [191.52; 223.81] | 58.00 | 248.00 ± 38.59 | 237.00 ± 15.75 | [217.12; 278.88] | 87.00 |
| Phosphate (P) | 0.7-1.5 mmol/L | 1.07 ± 0.20 | 1.00 ± 0.08 | [0.91; 1.22] | 0.50 | 0.95 ± 0.10 | 0.95 ± 0.04 | [0.87; 1.03] | 0.30 |
| Fructosamine | 120-290 μmo/L | 306.17 ± 18.73 | 305.00 ± 7.65 | [291.18; 321.16] | 46.00 | 256.67 ± 26.10 | 263.00 ± 10.66 | [235.78; 277.55] | 69.00 |
| Gamma-glutamyl transferase (GGT) | 6-22 IU/L | 13.50 ± 4.32 | 12.00 ± 1.77 | [10.04; 16.96] | 11.00 | 12.17 ± 5.00 | 11.00 ± 2.04 | [8.17; 16.16] | 13.00 |
| Glucose | 3.0-5.0 mmol/L | 4.87 ± 0.36 | 4.90 ± 0.15 | [4.58; 5.16] | 0.90 | 5.35 ± 0.29 | 5.35 ± 0.12 | [5.12; 5.58] | 0.70 |
| HCT | 32-42% | 39.17 ± 2.99 | 40.00 ± 1.22 | [36.77; 41.56] | 7.00 | 35.33 ± 2.80 | 34.50 ± 1.15 | [33.09; 37.58] | 8.00 |
| HGB | 90-190 g/L | 134.67 ± 11.86 | 139.00 ± 4.84 | [125.18; 144.16] | 29.00 | 121.33 ± 8.89 | 119.50 ± 3.63 | [114.22; 128.45] | 25.00 |
| Calcium (Ca) | 2.5-3.4 mmol/L | 2.87 ± 0.05 | 2.90 ± 0.02 | [2.83; 2.91] | 0.10 | 2.77 ± 0.08 | 2.75 ± 0.03 | [2.70; 2.83] | 0.20 |
| Potassium (K) | 2.8-4.5 mmol/L | 3.70 ± 0.48 | 3.85 ± 0.20 | [3.31; 4.09] | 1.40 | 3.72 ± 0.18 | 3.75 ± 0.07 | [3.57; 3.86] | 0.40 |
| Urea | 3.3-6.7 mmol/L | 3.73 ± 0.70 | 3.50 ± 0.29 | [3.17; 4.30] | 1.90 | 4.55 ± 0.70 | 4.45 ± 0.28 | [3.99; 5.11] | 1.80 |
| Chloride (Cl) | 90-108 mmol/L | 101.17 ± 1.33 | 101.00 ± 0.54 | [100.10; 102.23] | 3.00 | 107.33 ± 1.63 | 107.00 ± 0.67 | [106.03; 108.64] | 4.00 |
| Creatinine | 71-159 μmol/L | 135.17 ± 12.61 | 130.50 ± 5.15 | [125.08; 145.26] | 33.00 | 105.33 ± 10.13 | 102.50 ± 4.14 | [97.23; 113.44] | 26.00 |
| Lactate dehydrogenase (LDH) | 225-700 IU/L | 720.50 ± 166.22 | 690.00 ± 67.86 | [587.50; 853.50] | 405.00 | 827.00 ± 225.71 | 850.50 ± 92.15 | [646.39; 1007.61] | 618.00 |
| Magnesium (Mg) | 0.5-0.9 mmol/L | 0.64 ± 0.10 | 0.59 ± 0.04 | [0.55; 0.72] | 0.26 | 0.80 ± 0.03 | 0.80 ± 0.01 | [0.78; 0.82] | 0.08 |
| Sodium (Na) | 125-150 mmol/L | 137.95 ± 1.04 | 138.10 ± 0.42 | [137.12; 138.78] | 2.60 | 136.00 ± 1.17 | 135.80 ± 0.48 | [135.06; 136.94] | 3.10 |
| Sodium-to-potassium ratio |  | 37.92 ± 5.97 | 35.90 ± 2.44 | [33.14; 42.69] | 16.60 | 36.67 ± 2.03 | 36.45 ± 0.83 | [35.05; 38.29] | 4.40 |
| PLT | 50-300 G/L | 105.83 ± 18.41 | 102.50 ± 7.52 | [91.10; 120.57] | 49.00 | 112.83 ± 14.91 | 110.50 ± 6.09 | [100.91; 124.76] | 41.00 |
| RBC | 5-12 T/L | 7.65 ± 0.70 | 7.95 ± 0.28 | [7.09; 8.21] | 1.80 | 6.88 ± 0.74 | 7.00 ± 0.30 | [6.29; 7.48] | 2.20 |
| Total bilirubin | 7-60 μmol/L | 24.68 ± 6.87 | 23.30 ± 2.80 | [19.19; 30.18] | 19.80 | 30.57 ± 6.77 | 32.20 ± 2.77 | [25.15; 35.99] | 19.90 |
| Direct bilirubin | 1-10 μmol/L | 7.14 ± 0.86 | 7.10 ± 0.39 | [6.38; 7.90] | 2.40 | 7.92 ± 0.94 | 8.00 ± 0.47 | [7.00; 8.85] | 2.30 |
| Total cholesterol | 2.0-3.1 mmol/L | 2.52 ± 0.33 | 2.55 ± 0.14 | [2.25; 2.78] | 0.90 | 2.35 ± 0.26 | 2.40 ± 0.11 | [2.14; 2.56] | 0.70 |
| Total protein | 55-75 g/L | 66.78 ± 4.14 | 67.20 ± 1.69 | [63.47; 70.10] | 11.50 | 65.63 ± 4.79 | 65.80 ± 1.96 | [61.80; 69.47] | 13.40 |
| Triglycerides (TG) | 0.02-0.5 mmol/L | 0.51 ± 0.12 | 0.46 ± 0.05 | [0.42; 0.60] | 0.30 | 0.50 ± 0.22 | 0.52 ± 0.09 | [0.33; 0.68] | 0.63 |
| Iron (Fe) | 14.5-25 μmol/L | 34.40 ± 5.89 | 33.00 ± 2.41 | [29.68; 39.12] | 14.90 | 36.02 ± 13.49 | 35.10 ± 5.51 | [25.22; 46.81] | 37.40 |
| WBC | 5-10 G/L | 5.68 ± 0.40 | 5.65 ± 0.16 | [5.37; 6.00] | 1.10 | 5.33 ± 0.44 | 5.45 ± 0.18 | [4.98; 5.69] | 1.10 |
| **CBD group** |  |  |  |  |  |  |  |  |  |
| Alkaline phosphatase (ALP) | 147-261 IU/L | 266.86 ± 37.78 | 260.00 ± 14.28 | [238.87; 294.84] | 115.00 | 221.14 ± 28.55 | 211.00 ± 10.79 | [199.99; 242.29] | 87.00 |
| Glutamate dehydrogenase (GLDH) | 1-15 IU/L | 2.80 ± 0.84 | 3.00 ± 0.37 | [2.07; 3.53] | 2.00 | 3.00 ± 1.41 | 3.00 ± 0.58 | [1.87; 4.13] | 4.00 |
| Alanine aminotransferase (ALT) | 1-15 IU/L | 7.86 ± 1.57 | 8.00 ± 0.59 | [6.69; 9.02] | 5.00 | 13.86 ± 1.68 | 14.00 ± 0.63 | [12.62; 15.10] | 5.00 |
| Aspartate aminotransferase (AST) | 160-412 IU/L | 313.00 ± 40.39 | 315.00 ± 15.26 | [283.08; 342.92] | 118.00 | 318.57 ± 44.15 | 315.00 ± 16.69 | [285.86; 351.28] | 130.00 |
| Albumin | 26-41 g/L | 29.51 ± 1.71 | 30.30 ± 0.65 | [28.25; 30.78] | 5.10 | 32.86 ± 1.98 | 33.30 ± 0.75 | [31.39; 34.33] | 5.70 |
| Creatine kinase (CK) | 60-330 IU/L | 235.29 ± 34.56 | 221.00 ± 13.06 | [209.68; 260.89] | 96.00 | 289.00 ± 59.73 | 281.00 ± 22.57 | [244.75; 333.25] | 173.00 |
| Phosphate (P) | 0.7-1.5 mmol/L | 0.96 ± 0.11 | 0.90 ± 0.04 | [0.87; 1.04] | 0.30 | 0.99 ± 0.13 | 1.00 ± 0.05 | [0.89; 1.09] | 0.40 |
| Fructosamine | 120-290 μmol/L | 305.71 ± 20.42 | 308.00 ± 7.72 | [290.59; 320.84] | 64.00 | 249.86 ± 25.25 | 256.00 ± 9.54 | [231.15; 268.56] | 82.00 |
| Gamma-glutamyl transferase (GGT) | 6-22 IU/L | 14.29 ± 3.20 | 14.00 ± 1.21 | [11.92; 16.66] | 10.00 | 14.29 ± 4.11 | 15.00 ± 1.55 | [11.24; 17.33] | 12.00 |
| Glucose | 3.0-5.0 mmol/L | 4.84 ± 0.45 | 4.80 ± 0.17 | [4.51; 5.18] | 1.40 | 5.30 ± 0.33 | 5.30 ± 0.12 | [5.06; 5.54] | 1.00 |
| HCT | 32-42% | 36.43 ± 3.55 | 36.00 ± 1.34 | [33.80; 39.06] | 10.00 | 34.00 ± 2.89 | 35.00 ± 1.09 | [31.86; 36.14] | 8.00 |
| HGB | 90-190 g/L | 125.71 ± 12.50 | 126.00 ± 4.72 | [116.45; 134.97] | 33.00 | 117.14 ± 9.70 | 120.00 ± 3.67 | [109.95; 124.33] | 28.00 |
| Calcium (Ca) | 2.5-3.4 mmol/L | 2.89 ± 0.09 | 2.90 ± 0.03 | [2.82; 2.95] | 0.20 | 2.76 ± 0.13 | 2.80 ± 0.05 | [2.66; 2.85] | 0.30 |
| Potassium (K) | 2.8-4.5 mmol/L | 3.83 ± 0.17 | 3.80 ± 0.06 | [3.70; 3.95] | 0.50 | 3.80 ± 0.14 | 3.80 ± 0.05 | [3.70; 3.90] | 0.40 |
| Urea | 3.3-6.7 mmol/L | 4.14 ± 0.77 | 4.20 ± 0.29 | [3.57; 4.71] | 2.00 | 4.37 ± 0.55 | 4.30 ± 0.21 | [3.97; 4.78] | 1.70 |
| Chloride (Cl) | 90-108 mmol/L | 101.14 ± 2.04 | 101.00 ± 0.77 | [99.64; 102.65] | 6.00 | 107.29 ± 0.95 | 107.00 ± 0.36 | [106.58; 107.99] | 3.00 |
| Creatinine | 71-159 μmol/L | 140.14 ± 14.14 | 143.00 ± 5.34 | [129.67; 150.61] | 37.00 | 113.14 ± 10.88 | 115.00 ± 4.11 | [105.08; 121.21] | 31.00 |
| Lactate dehydrogenase (LDH) | 225-700 IU/L | 895.71 ± 246.06 | 922.00 ± 93.00 | [713.43; 1078.00] | 753.00 | 980.00 ± 256.43 | 978.00 ± 96.92 | [790.03; 1169.97] | 771.00 |
| Magnesium (Mg) | 0.5-0.9 mmol/L | 0.60 ± 0.09 | 0.57 ± 0.03 | [0.53; 0.66] | 0.25 | 0.78 ± 0.06 | 0.81 ± 0.02 | [0.73; 0.82] | 0.18 |
| Sodium (Na) | 125-150 mmol/L | 137.76 ± 1.11 | 138.00 ± 0.42 | [136.94; 138.58] | 3.60 | 135.93 ± 1.11 | 136.20 ± 0.42 | [135.11; 136.75] | 3.00 |
| Sodium-to-potassium ratio |  | 36.04 ± 1.54 | 36.30 ± 0.58 | [34.90; 37.18] | 4.00 | 35.80 ± 1.28 | 35.50 ± 0.48 | [34.85; 36.75] | 3.60 |
| PLT | 50-300 G/L | 116.14 ± 22.99 | 122.00 ± 8.69 | [99.11; 133.17] | 73.00 | 127.43 ± 17.08 | 132.00 ± 6.45 | [114.78; 140.08] | 48.00 |
| RBC | 5-12 T/L | 7.03 ± 0.81 | 7.10 ± 0.31 | [6.43; 7.63] | 2.40 | 6.53 ± 0.77 | 6.80 ± 0.29 | [5.96; 7.10] | 2.30 |
| Total bilirubin | 7-60 μmol/L | 21.77 ± 2.93 | 20.80 ± 1.11 | [19.60; 23.94] | 8.20 | 25.67 ± 2.67 | 25.80 ± 1.01 | [23.69; 27.65] | 8.10 |
| Direct bilirubin | 1-10 μmol/L | 7.37 ± 0.55 | 7.30 ± 0.21 | [6.97; 7.78] | 1.60 | 8.69 ± 0.79 | 9.00 ± 0.30 | [8.10; 9.27] | 2.20 |
| Total cholesterol | 2.0-3.1 mmol/L | 2.51 ± 0.20 | 2.40 ± 0.08 | [2.36; 2.67] | 0.50 | 2.37 ± 0.19 | 2.40 ± 0.07 | [2.23; 2.51] | 0.50 |
| Total protein | 55-75 g/L | 66.17 ± 3.29 | 67.90 ± 1.24 | [63.74; 68.61] | 8.40 | 64.60 ± 3.03 | 65.60 ± 1.15 | [62.35; 66.85] | 8.50 |
| Triglycerides (TG) | 0.02-0.5 mmol/L | 0.60 ± 0.07 | 0.62 ± 0.03 | [0.55; 0.65] | 0.19 | 0.74 ± 0.10 | 0.75 ± 0.04 | [0.66; 0.81] | 0.30 |
| Iron (Fe) | 14.5-25 μmol/L | 33.43 ± 5.26 | 34.50 ± 1.99 | [29.53; 37.33] | 17.00 | 39.67 ± 7.43 | 41.60 ± 2.81 | [34.16; 45.18] | 23.20 |
| WBC | 5-10 G/L | 6.10 ± 0.66 | 6.00 ± 0.25 | [5.61; 6.59] | 1.90 | 5.69 ± 0.84 | 5.80 ± 0.32 | [5.06; 6.31] | 2.60 |
