# Supplementary material for: Metabolic effects and pharmacokinetics of oral cannabidiol (CBD) in Connemara ponies following 21 days of treatment
Source: Front Vet Sci. 2026 May 14;13:1813917. doi: 10.3389/fvets.2026.1813917 (PMC13215832; doi:10.3389/fvets.2026.1813917)
Supplement: Supplementary file 2 [file Table_2.docx]

Table S2: Descriptive statistics of the blood glucose level during oral sugar test (OST) in the Control (n= 6) and the CBD (n= 7) group before and 24 hours after treatment. In the case of every measured variable the mean, standard deviation (SD), median, standard error (SE), 95% confidence interval and range (differences between the highest and lowest value) were calculated from the original dataset. The calculation did not exclude any ponies.

|  |  | **Glucose 60 min (mmol/L)** | | | | **Glucose 90 min (mmol/L)** | | | |
| --- | --- | --- | --- | --- | --- | --- | --- | --- | --- |
|  |  | **Mean±SD** | **Median±SE** | **95% CI** | **Range** | **Mean±SD** | **Median±SE** | **95% CI** | **Range** |
| **Treatment group** | **Time** |  |  |  |  |  |  |  |  |
| **Control group** | **Before treatment** | 7.20±0.98 | 7.45±0.40 | [6.42;7.98] | 2.8 | 7.72±1.18 | 8.0±0.48 | [6.77;8.66] | 3.3 |
|  | **After treatment** | 7.67±0.63 | 7.80±0.26 | [7.16;8.17] | 1.8 | 8.12±0.56 | 8.1±0.23 | [7.67;8.56] | 1.3 |
| **CBD group** | **Before treatment** | 7.96±0.77 | 7.80±0.29 | [7.39;8.53] | 2.1 | 8.41±1.08 | 8.0±0.41 | [7.61;9.21] | 3.2 |
|  | **After treatment** | 8.00±0.35 | 7.90±0.13 | [7.74;8.26] | 1.0 | 8.44±0.60 | 8.2±0.23 | [8.00;8.89] | 1.7 |
