# Supplementary material for: Metabolic effects and pharmacokinetics of oral cannabidiol (CBD) in Connemara ponies following 21 days of treatment
Source: Front Vet Sci. 2026 May 14;13:1813917. doi: 10.3389/fvets.2026.1813917 (PMC13215832; doi:10.3389/fvets.2026.1813917)
Supplement: Supplementary file 3 [file Table_3.docx]

Table S3: Descriptive statistics of the blood insulin level during oral sugar test (OST) in the Control (n= 6) and the CBD (n= 7) group before and 24 hours after treatment. In the case of every measured variable the mean, standard deviation (SD), median, standard error (SE), 95% confidence interval and range (differences between the highest and lowest value) were calculated from the original dataset. The calculation did not exclude any ponies.

|  |  | **Insulin 60 min (μIU/mL)** | | | | **Insulin 90 min (μIU/mL)** | | | |
| --- | --- | --- | --- | --- | --- | --- | --- | --- | --- |
|  |  | **Mean±SD** | **Median±SE** | **95% CI** | **Range** | **Mean±SD** | **Median±SE** | **95% CI** | **Range** |
| **Treatment group** | **Time** |  |  |  |  |  |  |  |  |
| **Control group** | **Before treatment** | 59.40±47.86 | 43.45±19.54 | [21.10;97.70] | 129.5 | 58.62±35.68 | 59.90±14.57 | [30.07;87.17] | 104.6 |
|  | **After treatment** | 60.07±33.52 | 52.10±13.68 | [33.25;86.89] | 88.9 | 54.45±30.45 | 43.15±12.43 | [30.08;78.82] | 78.7 |
| **CBD group** | **Before treatment** | 77.01±37.29 | 94.60±14.10 | [49.39;104.64] | 90.6 | 86.04±46.97 | 92.50±17.75 | [51.24;120.84] | 110.5 |
|  | **After treatment** | 92.97±34.37 | 97.70±12.99 | [67.51;118.43] | 95.2 | 91.57±29.26 | 102.00±11.06 | [69.90;113.24] | 78.9 |
